# Transcranial Red and Near Infrared Light Transmission in a Cadaveric Model

**DOI:** 10.1371/journal.pone.0047460

**Published:** 2012-10-15

**Authors:** Jared R. Jagdeo, Lauren E. Adams, Neil I. Brody, Daniel M. Siegel

**Affiliations:** 1 Department of Dermatology, State University of New York Downstate Medical Center, Brooklyn, New York, United States of America; 2 Dermatology Service, Sacramento Veterans Affairs Medical Center, Mather, California, United States of America; 3 Department of Dermatology, University of California Davis, Sacramento, California, United States of America; 4 Dermatology Service, Brooklyn Veterans Affairs Medical Center, Brooklyn, New York, United States of America; MGH, MMS, United States of America

## Abstract

**Background and Objective:**

Low level light therapy has garnered significant interest within the past decade. The exact molecular mechanisms of how red and near infrared light result in physiologic modulation are not fully understood. Heme moieties and copper within cells are red and near infrared light photoreceptors that induce the mitochondrial respiratory chain component cytochrome C oxidase, resulting in a cascade linked to cytoprotection and cellular metabolism. The copper centers in cytochrome C oxidase have a broad absorption range that peaks around 830 nm. Several *in vitro* and *in vivo* animal and human models exist that have demonstrated the benefits of red light and near infrared light for various conditions. Clinical applications for low level light therapy are varied. One study in particular demonstrated improved durable functional outcomes status post-stroke in patients treated with near infrared low level light therapy compared to sham treatment [1]. Despite previous data suggesting the beneficial effect in treating multiple conditions, including stroke, with low level light therapy, limited data exists that measures transmission in a human model.

**Study Design/Materials and Methods:**

To investigate this idea, we measured the transmission of near infrared light energy, using red light for purposes of comparison, through intact cadaver soft tissue, skull bones, and brain using a commercially available LED device at 830 nm and 633 nm.

**Results:**

Our results demonstrate that near infrared measurably penetrates soft tissue, bone and brain parenchyma in the formalin preserved cadaveric model, in comparison to negligible red light transmission in the same conditions.

**Conclusion:**

These findings indicate that near infrared light can penetrate formalin fixed soft tissue, bone and brain and implicate that benefits observed in clinical studies are potentially related to direct action of near infrared light on neural tissue.

## Introduction

Infrared (IR) light is invisible to the naked eye, with wavelengths between 750 nanometers (nm) and 1 millimeter (mm) [2]. The shorter limit of these wavelengths borders on the visible light spectrum, and the longer limit is adjacent to microwaves in the light spectrum [2]. Within the infrared spectrum, there are five distinct categories: near infrared (IR-A, 750–1400 nm), short-wavelength infrared (IR-B, 1.4–3 micrometers (µm)), mid-wavelength infrared (IR-C, 3–8 µm), long-wavelength infrared (also referred to as IR-C, 8–15 µm), and far infrared region (15 µm to 1 mm) [2]. Recent studies have suggested the use of infrared light as therapy for an array of diseases, including dermatologic conditions, photoaging, wound healing, alopecia areata and temporal mandibular joint (TMJ) pain [3], [4], [5], [6], [7], [8], [9]. Near infrared light transmitted by light emitting diodes (LED) has been found to increase *in vitro* cell growth of mouse fibroblasts, rat osteoblasts, rat skeletal muscle cells, and normal human epithelial cells [10]. Near infrared light has also been found to accelerate wound healing in both mice and humans [4], [5], [6], [10], possibly via upregulation of transforming growth factor (TGF)-beta 1 and matrix metalloproteinase (MMP)-2 content [4].

### Near Infrared Light and Neurological Studies

Neurologically, infrared light has been reported to demonstrate benefit in diabetic peripheral neuropathy, as well as in strokes. In a double-blind, randomized, placebo-controlled study, diabetic patients treated with a near infrared device experienced an improvement in sensation in the lower extremities (as measured by the 5.07 and 6.65 Semmes Weinstein monofilament) and decrease in neuropathic symptoms (as measured by a modified Michigan Neuropathy Screening Instrument) [11]. Furthermore, while 90% of patients initially reported balance impairment, only 17% did so after treatment [11].

**Figure 1:**
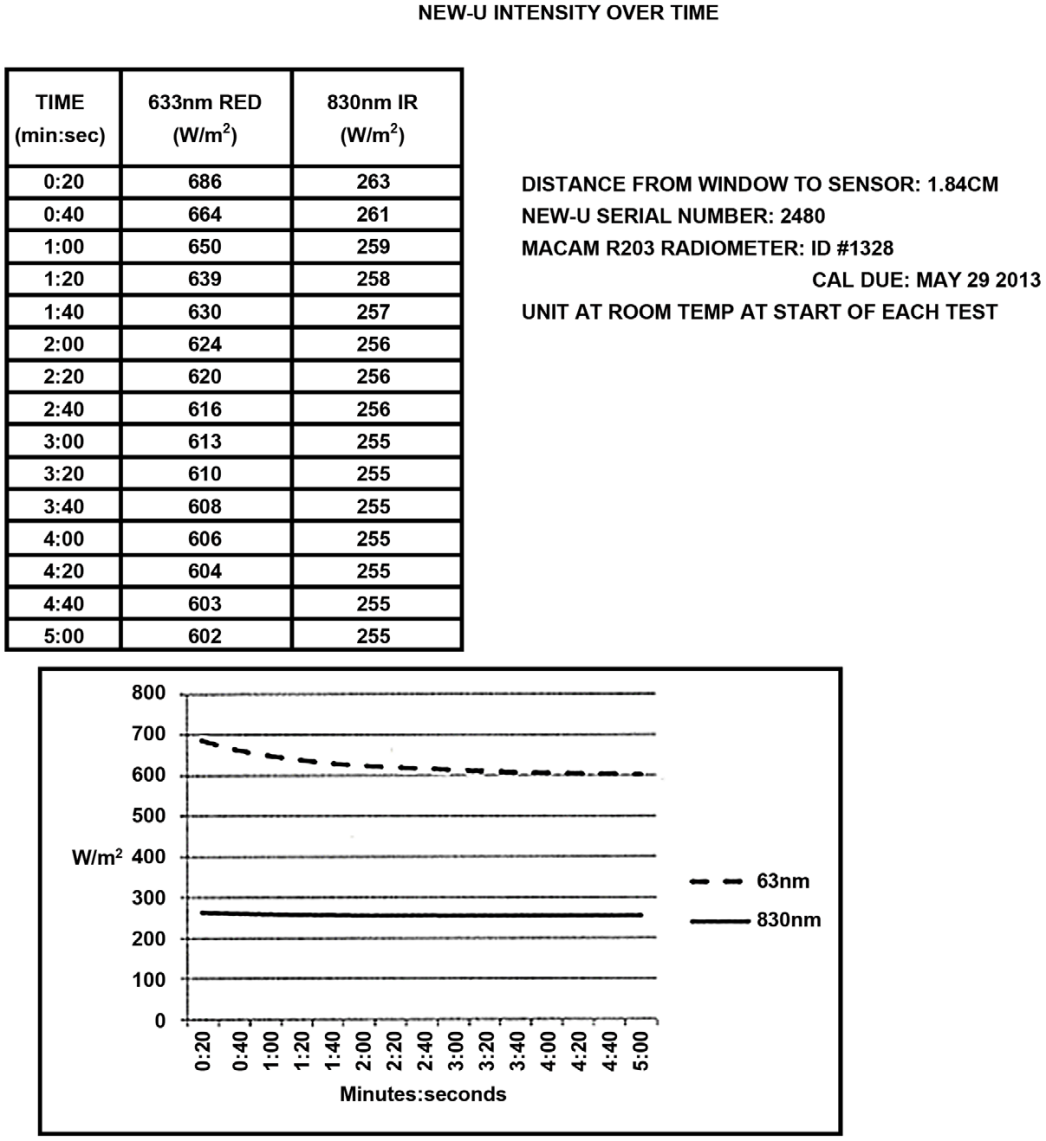
LED Stability Performance for Red and Near Infrared Light over 5 minutes. Output of red light and near infrared light from the LED device is relatively stable over time.

In a 2007 study of embolic strokes in rabbits, behavioral analysis 48 hours after embolization showed improvement in rabbits treated 6 hours after embolization with pulse wave near infrared light therapy at a frequency of 300 µs at 1 kHz or at a frequency of 2 ms at 100 Hz [12]. Improvement was also seen in those treated at 12 hours post-embolization time with pulse wave near infrared light therapy at a frequency 2 ms at 100 Hz, but no improvement was seen with continuous wave near infrared light therapy at either time point [12].

**Figure 2:**
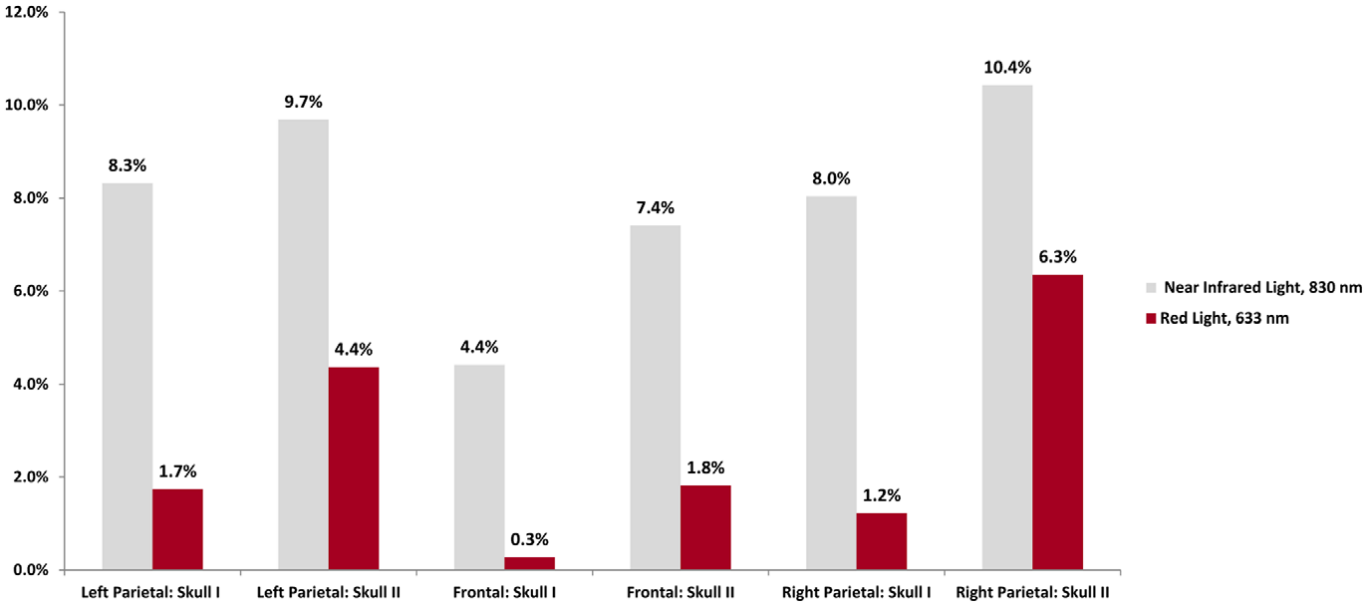
Percent Penetrance of Light through Coronal Sections of Cadaver Skull, Bone Only. Near infrared light measurably penetrates cadaver skull, as compared to red light.

**Table 1:** Transmission of Near Infrared and Red Light through Cadaver Skulls in Coronal Sections.

	Near Infrared Light, 830 nm (milliwatts/cm^2^)		Red Light, 633 nm (milliwatts/cm^2^)	
	Skull I	Skull II	Skull I	Skull II
Air only, at a distance of 5 mm	35.1		72.6	
Left Parietal Skull	2.92	3.40	1.265	3.17
Frontal Skull	1.55	2.60	0.20	1.32
Right Parietal Skull	2.82	3.66	0.89	4.61

Similar improvement has been shown in humans undergoing stroke. The NeuroThera Effectiveness and Safety Trial-1 (NEST-1) was a prospective, double-blind study of 120 ischemic stroke patients randomized to receive transcranial infrared laser treatment within 24 hours of stroke or sham placebo treatment. Seventy percent of treated patients had successful outcomes (measured by the National Institutes of Health Stroke Scale (NIHSS), and defined as complete recovery at day 90 or a decrease in NIHSS score by at least 9 points), compared to 51% of controls [1]. In a follow-up 2009 study, NEST-2, of 660 patients with acute ischemic stroke, 36.3% of patients treated with transcranial near infrared laser technology within 24 hours had a favorable outcome (defined by a 90 day score of 0–2 on the modified Rankin Scale), as compared to 30.9% [13]. Although this finding was not statistically significant, it does suggest a favorable outcome with the treatment [13]. There was no difference in mortality or serious adverse effects between the treated and untreated groups [13].

**Figure 3:**
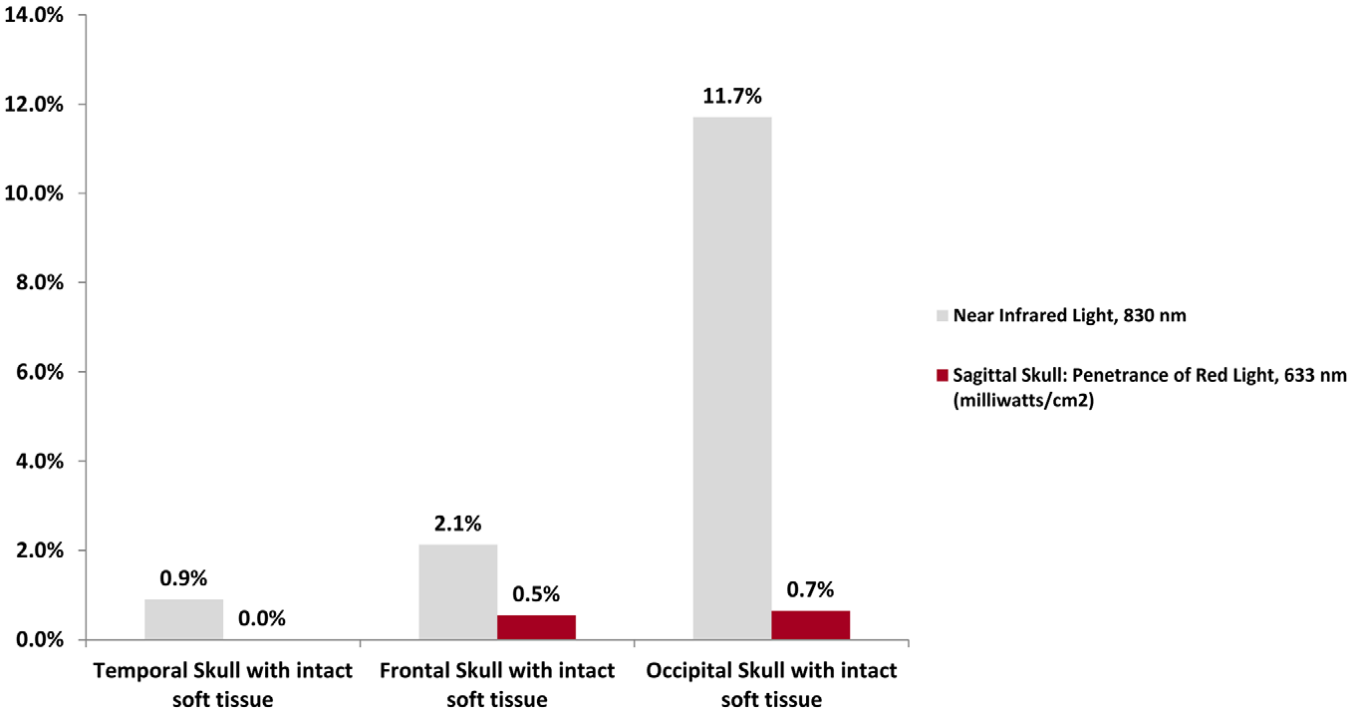
Percent Penetrance of Light through Sagittal Sections of Cadaver Skull with Intact Soft Tissue. Near infrared light measurably penetrates cadaver skull with intact soft tissue, as compared to red light.

**Table 2:** Transmission of Near Infrared and Red Light through Sagittally Cut Intact Cadaver Head and Intact Shoulder and Temporomandibular Joint.

	Near Infrared Light, 830 nm (milliwatts/cm^2^)	Red Light, 633 nm (milliwatts/cm^2^)
Air only, at a distance of 10 mm	33.3	67.5
Temporal Skull with overlying soft tissue intact	0.30	<0.001
Frontal Skull with overlying soft tissue intact	0.71	0.37
Occipital with overlying soft tissue intact	3.90	0.44

### A Systemic Mechanism of Action via Blood Irradiation?

In terms of mechanism of action, a systemic effect may be responsible for some of the therapeutic value of infrared light. Interestingly, the growth promoting activity of the entire circulating blood is enhanced by local infrared irradiation [14], [15]. Blood from human volunteers whose sacral skin had been irradiated with visible and infrared polarized light was found to increase proliferation of *in vitro* keratinocytes [14]. Similar results were obtained with blood that was irradiated *in vitro* [14]. The authors hypothesized that transcutaneous photomodification of a small amount of blood in superficial skin vessels may lead to rapid rise of the growth promoting activity of the entire circulated blood, possibly via release of growth factors from blood cells [15]. One factor that may be released is nitric oxide (NO). Near-infrared light irradiation has been shown to increase nitric oxide production in cultured rat and mouse cardiomyocytes, and protect them from injury at the onset of reoxygenation following hypoxia [16]. NO has a number of effects on cells, including a role in apoptotic pathways, and may promote or antagonize apoptosis depending on its concentration and the cellular redox state [16].

**Figure 4:**
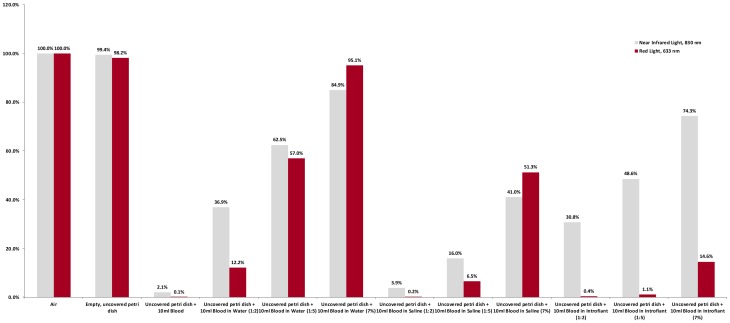
Percent Penetrance of Light through Various Concentrations of Blood. Blood attenuates the transmission of both near infrared and red lights. When blood was diluted in normal saline to a concentration of 7%, representing physiologic conditions, transmission of near infrared light was decreased to 41%.

**Table 3:** Transmission of Near Infrared and Red Light through Various Concentrations of Blood.

Wavelength	Media Combinations											
	Air only	Petri dish only	Blood	Hemolyzed Blood in H_2_O			Non-hemolyzed Blood in Saline			Blood in Introfiant		
				1∶2	1∶5	7% Blood	1∶2	1∶5	7% Blood	1∶2	1∶5	7% Blood
Near Infrared Light, 830 nm (milliwatts/cm^2^)	30.34	30.16	0.625	11.2	18.96	25.76	1.192	4.86	12.44	9.352	14.74	22.54
Red Light, 633 nm (milliwatts/cm^2^)	59.40	58.32	0.0363	7.27	33.85	56.50	0.122	3.88	30.46	0.218	0.666	8.686

### Aims of this Study

Despite prior research that has shown beneficial effects in treating stroke patients with infrared irradiation, limited data exists that demonstrates the ability of infrared light to pass through the soft tissue and bone of the skull. In fact, in dermatology textbooks, 600–1064 nm wavelength light is commonly depicted as penetrating no further than the dermis [17]. As dermatologists aware of the potential dermatologic uses for near-infrared light, we were intrigued by the findings of NEST-1 and NEST-2, and debated whether the findings were due to direct or indirect effects of the light. Taking into account the dermatologic benefits that have been seen with infrared light, and the finding that local skin irradiation leads to change in the circulating blood, we theorized that perhaps some of the beneficial effects seen in stroke patients are indirect, secondary to dermatologic or hematologic modulation. To investigate this idea, we measured the passage of infrared light through cadaver skull bones, sectioned cadaver skulls with intact soft tissue, *in vivo* human cheek, and *in vivo* human hand. For comparison, we also analyzed the passage of red light through these materials, as red light is also used therapeutically for multiple medical conditions, including wound repair, dermatologic diseases, neurologic damage, blood disorders, musculoskeletal complications, and inflammation [18]. Water, saline, cadaver fixative, and blood at various dilutions were also evaluated.

**Figure 5:**
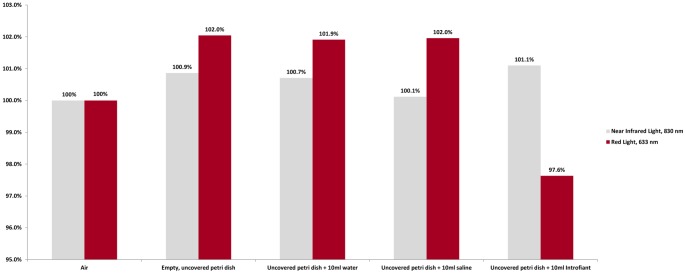
Percent Penetrance of Light through Various Media. Water, saline, and cadaver fixative (Introfiant) have little effect on the transmission of near infrared and red lights.

**Table 4:** Transmission of Near Infrared and Red Light through Various Media.

Vessel, Media	Near Infrared Light, 830 nm (milliwatts/cm^2^)	Red Light, 633 nm (milliwatts/cm^2^)
Air only	25.45	61.21
Empty, uncovered petri dish	25.67	62.46
Uncovered petri dish +10 mL water	25.63	62.38
Uncovered petri dish +10 mL saline	25.48	62.41
Uncovered petri dish +10 mL Introfiant	25.73	59.76

## Methods

### Ethics

Informed consent was not obtained for use of cadaveric samples, as these bodies had been donated to medical scientific study, including dissection, by the deceased. The cadaver skulls and tissues belonged to the State University of New York Downstate Medical Center anatomy lab. No tissue dissection was performed, and only previously dissected and sectioned skulls were used. The research study protocol was reviewed and approved by the director of the State University of New York Downstate Medical Center anatomy lab, as the modifying element of the study consisted of non-invasive light based exposure and measurements, within the scope of the cadaveric donation to biomedical science.

**Figure 6:**
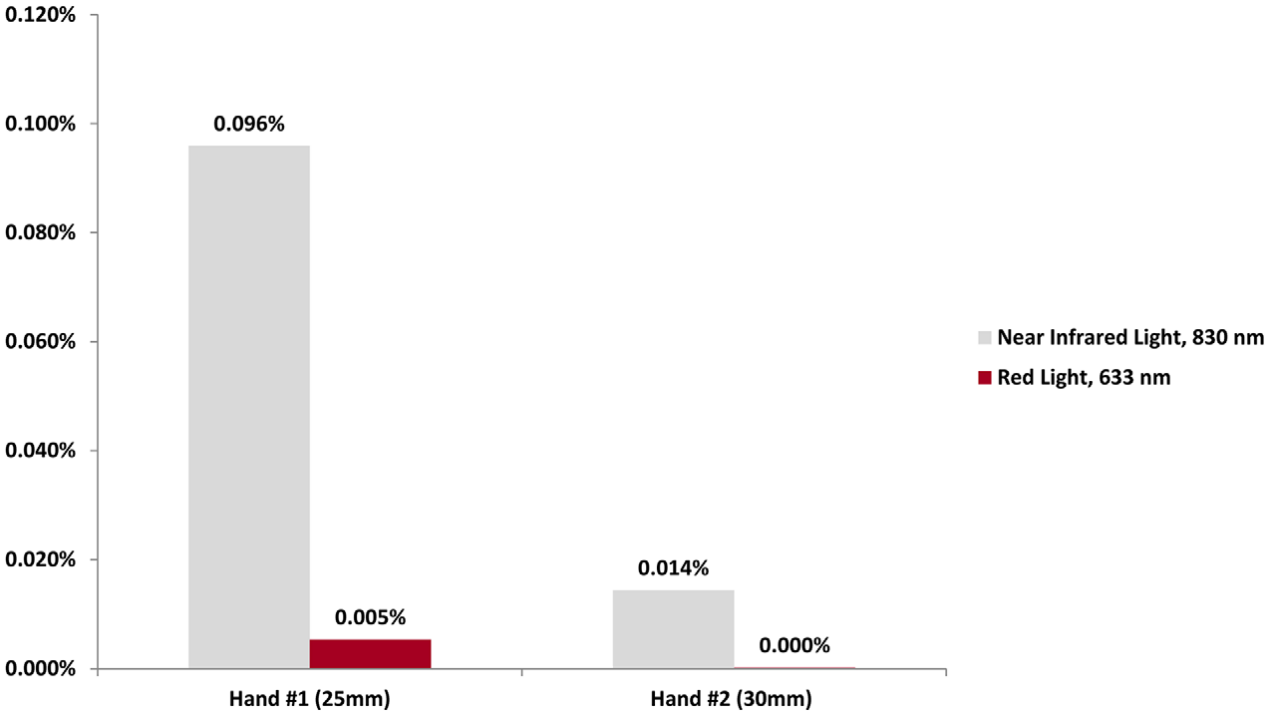
Percent Penetrance of Light through Human Hand *in vivo.* Transmission of near infrared light through a human hand is low, but quantifiable, and is greater than red light transmission.

**Table 5:** Transmission of Near Infrared and Red Light through Hands.

	Near Infrared Light, 830 nm (milliwatts/cm^2^)	Red Light, 633 nm (milliwatts/cm^2^)
Air only, at distance of 25 mm	27.1	56.0
Hand #1 (25 mm thick)	0.026	0.003
Air only, at distance of 30 mm	25.0	51.4
Hand #2 (30 mm thick)	0.0036	0.0001

Ethics approval was not sought from our institutional review board for use of human subjects, because the authors themselves served as the subjects of the experiments, and the most invasive procedure was a single blood draw. Neither written nor verbal informed consent was obtained from the participants, as the participants were the authors, and would have administered the consent to themselves.

**Figure 7:**
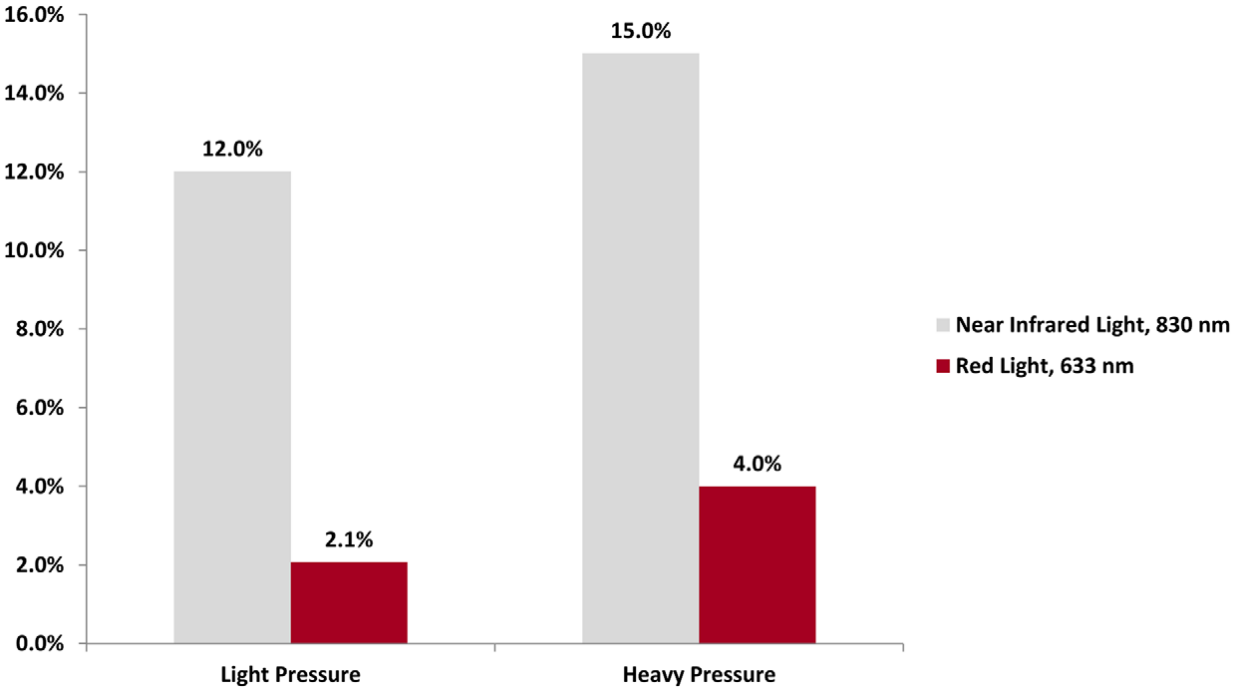
Percent Penetrance of Light through Human Cheek *in vivo.* Transmission of near infrared light through a human cheek is significant, and is greater than transmission of red light.

**Table 6:** Transmission of Near Infrared and Red Light through a Human Cheek.

	Near Infrared Light, 830 nm (milliwatts/cm^2^)	Red Light, 633 nm (milliwatts/cm^2^)
Air only, at a distance of 10 mm	33.3	67.5
Cheek, Light Pressure Applied	4.0	1.4
Cheek, Heavy Pressure Applied	5.0	2.7

### Transmission of Near Infrared and Red Light through Cadaver Skulls

The transmission of near infrared light and red light through cadaveric skull and intact cadaver sagittally sectioned head was measured using a Macam, now called Irradian, Radiometer (Model R203) with a 1.5 cm diameter sensor irradiance filter ring detector (RFF Cos-112). The light source used was an Omnilux New-U hand held device with a 4.7 cm ×6.1 cm rectangular emitting aperture (kindly provided by Photomedex) and measurements were recorded of the transmission of near infrared light and red light through two coronally sectioned cadaver skulls. The penetrance was recorded through the frontal, left parietal, and right parietal skull. This process was repeated with a sagittally cut cadaver head with intact soft tissue. In this case, the penetrance of near infrared and red light was recorded through the frontal, temporal, and occipital skull. LED stability performance for red light and near infrared light was quantified over a 5 minute period and is presented in Figure 1.

### Transmission of Near Infrared and Red Light through Various Concentrations of Blood

The penetrance of near infrared and red light through human blood was measured in petri dishes. A Macam R203 Radiometer was fixed in place 1.84 cm above a non-mobile Omnilux New-U hand held light source. The passage of red light and near infrared light across this distance was measured. Then, the penetration of red light and near infrared light was measured when an uncovered Pyrex 60×15 mm petri dish, with its vertical column wrapped with duct tape to minimize ambient light contamination, was placed on top of the light source. Following this, 10 mL of human blood was placed in the petri dish, and the transmission of red light and near infrared light were measured. Next, serial dilutions of blood were made with water, saline (Baxter), and Introfiant (Dodge), the fixative used in this cadaver. Blood was diluted 1∶2, 1∶5, and then to 7% in each of these media. The concentration 7% was chosen because blood accounts for approximately 7% of body weight [19]. For each dilution, 10 mL were placed in a petri dish, and the transmission of red light and infrared light across the petri dish were measured.

### Testing of Media Controls

To clarify the effect that embalming fluid may have had on the penetrance of near infrared and red light through the cadaver tissue, we measured the passage of near infrared light and red light through water, saline, and Introfiant. A Macam R203 Radiometer was fixed in place 1.84 cm above an Omnilux New-U hand held light source, which was also fixed in place. The passage of red light across this distance was measured. Then, the penetration of red light was measured when an uncovered Pyrex 60×15 mm petri dish, with its vertical column wrapped with duct tape to minimize ambient light contamination, was placed on top of the light source. Following this, 10 mL of water was placed in the dish, forming a column of liquid 4.95 mm high, and the penetrance of red light was measured. This was repeated with 10 mL of 0.9% sodium chloride, and then 10 mL of Introfiant. The same measurements were repeated using infrared light.

### Transmission of Near Infrared and Red Light through a Human Hand *in Vivo*

In order to obtain data about *in vivo* penetration of near infrared and red light through human tissue, the penetrance through two human hands was measured. First, the passages of near infrared and red light across 25 mm and 30 mm were measured. Following this, the passage of near infrared and red light across two different hands at midpalm level was measured. The first hand was 25 mm in thickness and the second hand was 30 mm in thickness.

### Transmission of Near Infrared and Red Light through a Human Cheek *in Vivo*

To follow up on the data obtained from the human hands, the penetrance of near infrared and red light through a human cheek was measured. The light meter was inserted into the mouth, and positioned with the sensor against the cheek. The light source was placed against the cheek from outside the mouth. First measurements of the penetrance of near infrared and red light were taken with light pressure applied. Then the measurements were repeated with the meter and light source pressed together with heavy pressure, to minimize blood flow through the area. It is possible that the pressure might have decreased the distance the LED-generated light had to penetrate and caused the light source to get closer to the radiometer as the tissue was less thick.

## Results

Results are presented as absolute values in milliwatts/cm^2^, as well as in percentages. Relative penetration percentage values were calculated using the following formula:
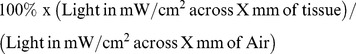



### Transmission of Near Infrared and Red Light through Cadaver Skulls

The results of the transmission of light through cadaver skulls in coronal section are presented in Table 1 in absolute values and in Figure 2 in relative penetration percentages. With the light source and light meter placed 5 mm away from each other (the approximate thickness of skull bone), 35.1 mW/cm^2^ of near infrared light and 72.6 mW/cm^2^ of red light reached the light sensor. These values served as baseline for the percent penetrance calculated.

The absolute values of the transmission of light through an intact cadaver head with soft tissue in sagittal section are presented in Table 2, and the relative penetration percentages of light transmission are shown in Figure 3. At 10 mm (the approximate thickness of cadaver skull with intact soft tissue), 33.3 mW/cm^2^ of near infrared and 67.5 mW/cm^2^ of red light penetrate air.

### Transmission of Near Infrared and Red Light through Various Concentrations of Blood

The results of the transmission of light through various concentrations of blood are shown in Table 3 for absolute values, and in Figure 4 for relative penetration values. With the light source and light meter fixed at a distance of 1.84 cm, 30.34 mW/cm^2^ of near infrared light and 59.40 mW/cm^2^ of red light penetrated air.

### Testing of Media Controls

The results of the transmission of light through various media are presented in Table 4 in absolute numbers, and in Figure 5 in relative values. With the light source and light meter fixed at a distance of 1.84 cm, 25.45 mW/cm^2^ of near infrared light and 61.21 mW/cm^2^ of red light reached the light source. We note that these differences are slight and may be attributed to power fluctuations or other causes, such as handling of instruments or samples.

### Transmission of Near Infrared and Red Light through a Human Hand *in Vivo*

The results of the penetrance of light through two different hands at midpalm level are presented in Table 5 in absolute values and in Figure 6 in percentages. The transmission of light across 25 mm of air (the thickness of hand #1) was 27.1 mW/cm^2^ for near infrared light and 56.0 mW/cm^2^ for red light. The transmission of light across 30 mm of air (the thickness of hand #2) was 25.0 mW/cm^2^ for near infrared light, and 51.4 mW/cm^2^ for red light.

### Transmission of Near Infrared and Red Light through a Human Cheek *in Vivo*

The results of the penetrance of light through a human cheek in absolute values are presented in Table 6, and Figure 7 for the results as relative penetration values. The transmission of light across 10 mm of air (the approximate thickness of a human cheek) was 33.3 mW/cm^2^ for near infrared light, and 67.5 mW/cm^2^ for red light.

## Discussion

These findings demonstrate that near infrared light measurably penetrates soft tissue, bone and brain parenchyma in the formalin preserved cadaveric model, in comparison to negligible red light transmission in the same conditions. There is usually a tissue color change that occurs over time from fresh fixation in formalin to permanent fixation in formalin. There is no blood in cadavers. The blood is drained and replaced with fixative. We used the human blood to account for another factor that could reduce the penetrance to the brain in vivo [20]. Limited data exists regarding the penetration of light of various wavelengths in human cadaveric models, but to our knowledge, no studies have taken into account the effect of fixative or blood on the penetration of light in cadaveric human models [21]. This study demonstrates that blood attenuates the transmission of light. However, transmission of near infrared light through an *in vivo* human cheek is significant. This is important, as the structure of the human cheek is similar to that of the scalp, in terms of soft tissue composition, thickness and vascular supply. We measured the thickness of the cheek to be approximately 10 mm, and the average living human scalp is approximately 5 to 6 mm thick [22]. However, as tissue thickness increases and when bones and an active vascular supply are present, as with the human hand *in* vivo, light penetration decreases, but remains quantifiable when near infrared light is used. The results suggest that benefits observed in clinical studies may be related to direct action of near infrared light on neural tissue, and that this action may only require very low levels of irradiance. An indirect effect cannot be excluded.

The major mechanism hypothesized to account for the direct therapeutic value of infrared light irradiation, especially in the brain, is increased adenosine triphosphate (ATP) formation after energy absorption by mitochondria. The majority of energy used by neurons is for membrane repolarization after depolarization (and thus for action potentials), as compared to protein synthesis and other cell functions [23]. Thus, during strokes, increasing ATP formation in neurons may enhance neuronal function, leading to better outcomes. ATP is also needed for all cellular activity, and to generate enzymes involved in cell survival, reproduction, and repair.

Hemoglobin, myoglobin, and cytochrome C oxidase are the three known major photoacceptors of near infrared light in mammalian tissue, and of these, only cytochrome C is implicated in energy production [24]. About 50% of near infrared light is absorbed by mitochondrial chromophores, specifically cytochrome C oxidase, which is part of the electron transport chain that is responsible for generation of ATP [25].

Cytochrome C oxidase is the terminal enzyme of the electron transport chain, ultimately responsible for creating an electrochemical potential across the inner mitochondrial membrane, which drives the production of ATP [26]. The enzyme is structurally large and complex, and possible absorbing chromophores include two heme moieties and two copper sites (Cu_A_ and Cu_B_) [26]. Analysis of the action spectrum for cellular proliferation following laser photoirradiation and spectroscopic data on cytochrome C oxidase has suggested that the majority of photoabsorption is via oxidized Cu_A_ (825 nm), reduced Cu_B_ (760 nm), oxidized Cu_B_ (680 nm), and reduced Cu_A_ (620 nm) [26].

Our results suggest that very low densities of light may reach the brain when near infrared light is applied to the skull of stroke patients. This direct irradiation may be sufficient to change mitochondrial and neural activity [27]. In previous studies treating strokes in rabbits and rats an 808 nm diode laser was set to give a power density of 7.5 mW/cm^2^ at brain level [12], [28]. Our measured irradiances through coronal sections of cadaver subjects are similar, albeit slightly lower. However, irradiance through frontal and temporal regions of a sagittally sectioned cadaver are approximately 10-fold lower, suggesting that placement of the light source can have a significant impact on the irradiance at the brain level. The average irradiance of infrared light through coronal and sagittal cadaver sections in our study is 2.43 mW/cm2. The NEST-1 trial was designed to deliver 1 Joule/cm^2^ to the entire surface of the cortex by treating 20 predetermined sites on the scalp for 2 minutes each [1]. Notably, previous trials with rabbits and rats delivered a similar energy density, 0.9 Joule/cm^2^ [12], [28]. Based upon our findings, in order to obtain 1 Joule/cm^2^ over the entire cortex surface, each site would need to be treated for an average of 6.86 minutes. More importantly, as our study illustrates, treatment times should be calculated based upon penetrance at various locations on the skull, as irradiance can vary significantly. It is important to note that the estimated energy density reaching the cortex of the patients in NEST-1, in which a coherent light source was used, was 1 J/cm^2^, which is much lower than the energy levels we observed using a noncoherent light source [1], [17].

Noncoherent versus coherent light penetrance has been the subject of some studies, and mathematical simulations, for example Monte-Carlo simulation, may provide insight into the ability of different light sources, such as laser compared to light-emitting diode, at the same wavelength to penetrate tissue [1], [29], [30]. Yet, recent studies, both *in vitro* and *in vivo*, have shown that low levels of infrared light can exert effects on neural tissue. In a 2009 study, a 670 nm laser with a low peak irradiance output of 3 mW/cm^2^ and a low dose of 0.45 mJ/cm^2^ was found to stimulate nerve growth factor-induced neurite elongation *in vitro*, and stabilize mitochondria membrane potential in neurons exposed to H_2_O_2_ [31]. Similarly, in a 2008 *in vivo* study in pigmented rats, 633 nm light emitting diode treatment with power density of 2 mW/cm^2^ was applied via two LED arrays (each of 44 LEDs) located 3.8 cm above the subjects' heads for 30 minutes [32]. The treatment increased whole-brain cytochrome oxidase and superoxide dismutase activities in a dose-dependent manner, and prevented the decrease in visual function induced by administration of rotenone, a mitochondrial complex I inhibitor [32].

Near infrared light measurably penetrates soft tissue, bone, and brain parenchyma in a formalin preserved cadaver specimen, and also penetrates the full thickness of human cheek *in vivo*. These findings support the hypothesis that direct irradiation of brain tissue may responsible for improved outcomes observed in stroke patients, although an indirect effect cannot be excluded. Since blood appreciably decreases the penetrance of light, it seems that direct effects observed may only require low levels of irradiance and low energy densities. Coupled with the ongoing research concerning photoabsorption, cytochrome C oxidase, and ATP generation, our findings provide a foundation for further investigation of the effects of near infrared light of the brain status post-stroke.
